# *Neisseria gonorrhoeae* lipooligosaccharide glycan epitopes recognized by bactericidal IgG antibodies elicited by the meningococcal group B-directed vaccine, MenB-4C

**DOI:** 10.3389/fimmu.2024.1350344

**Published:** 2024-02-19

**Authors:** Yih-Ling Tzeng, Soma Sannigrahi, Ray Borrow, David S. Stephens

**Affiliations:** ^1^Division of Infectious Diseases, Department of Medicine, Emory University School of Medicine, Atlanta, GA, United States; ^2^Meningococcal Reference Unit, UK Health Security Agency, Manchester Royal Infirmary, Manchester, United Kingdom; ^3^Department of Microbiology and Immunology, Emory University School of Medicine, Atlanta, GA, United States

**Keywords:** *Neisseria meningitidis*, lipooligosaccharide (LOS), meningococcal vaccine, IgG, glycan epitope, gonorrhea (*Neisseria gonorrhoeae*), bactericidal, endotoxin

## Abstract

**Introduction:**

Outer membrane vesicles (OMVs) of *Neisseria meningitidis* in the group B-directed vaccine MenB-4C (Bexsero^R^) protect against infections with *Neisseria gonorrhoeae*. The immunological basis for protection remains unclear. *N. meningitidis* OMV vaccines generate human antibodies to *N. meningitidis* and *N. gonorrhoeae* lipooligosaccharide (LOS/endotoxin), but the structural specificity of these LOS antibodies is not defined.

**Methods:**

Ten paired human sera obtained pre- and post-MenB-4C immunization were used in Western blots to probe *N. meningitidis* and *N. gonorrhoeae* LOS. Post-MenB-4C sera (7v5, 19v5, and 17v5), representing individual human variability in LOS recognition, were then used to interrogate structurally defined LOSs of *N. meningitidis* and *N. gonorrhoeae* strains and mutants and studied in bactericidal assays.

**Results and discussion:**

Post-MenB-4C sera recognized both *N. meningitidis* and *N. gonorrhoeae* LOS species, ~10% of total IgG to gonococcal OMV antigens. *N. meningitidis* and *N. gonorrhoeae* LOSs were broadly recognized by post-IgG antibodies, but with individual variability for LOS structures. Deep truncation of LOS, specifically a *rfa*K mutant without *α*-, *β*-, or *γ*-chain glycosylation, eliminated LOS recognition by all post-vaccine sera. Serum 7v5 IgG antibodies recognized the unsialyated L1 *α*-chain, and a 3-PEA-HepII or 6-PEA-HepII was part of the conformational epitope. Replacing the 3-PEA on HepII with a 3-Glc blocked 7v5 IgG antibody recognition of *N. meningitidis* and *N. gonorrhoeae* LOSs. Serum 19v5 recognized lactoneotetrose (LNT) or L1 LOS-expressing *N. meningitidis* or *N. gonorrhoeae* with a minimal *α*-chain structure of Gal-Glc-HepI (L8), a 3-PEA-HepII or 6-PEA-HepII was again part of the conformational epitope and a 3-Glc-HepII blocked 19v5 antibody binding. Serum 17v5 LOS antibodies recognized LNT or L1 *α*-chains with a minimal HepI structure of three sugars and no requirement for HepII modifications. These LOS antibodies contributed to the serum bactericidal activity against *N. gonorrhoeae*. The MenB-4C vaccination elicits bactericidal IgG antibodies to *N. gonorrhoeae* conformational epitopes involving HepI and HepII glycosylated LOS structures shared between *N. meningitidis* and *N. gonorrhoeae.* LOS structures should be considered in next-generation gonococcal vaccine design.

## 1 Introduction

Gonorrhea, a sexually transmitted infection caused by *Neisseria gonorrhoeae*, is an increasing global health concern. The World Health Organization estimated 82.4 million new gonorrhea cases among adolescents and adults worldwide in 2020. In the USA, approximately 1.6 million new gonococcal infections per year were estimated by the US Centers for Disease Control and Prevention, and gonorrhea is the second most reported bacterial communicable disease (1). The rise of multidrug-resistant gonorrhea (2, 3) suggests that a vaccine with even partial effectiveness against gonorrhea would provide a substantial public health benefit.

Gonococcal vaccine development has been challenging due to the lack of defined immune protection correlates and unclear mechanisms of protective immunity against gonococcal infections. However, declines in the incidence of gonorrhea, in contrast with other sexually transmitted infections, followed the implementation of *Neisseria meningitidis* outer membrane vesicles (OMV)-based vaccines in Norway, Cuba, and Canada (4–6). Furthermore, a 2017 retrospective casecontrol study assessed vaccine effectiveness against gonorrhea among young adults who received the meningococcal MeNZB vaccine (OMV vaccine derived from the New Zealand group B outbreak strain NZ98/254) and found an estimated vaccine effectiveness against gonorrhea of 31% after adjustment (7). Recently, additional retrospective studies reported the effectiveness of a broadly licensed *N. meningitidis* serogroup B meningococcal vaccine (MenB-4C/Bexsero) against gonorrhea. US data from New York City and Philadelphia showed that, compared to no vaccination, individuals who received two doses and one dose of MenB-4C had 40% and 26% effectiveness, respectively, in preventing gonorrhea (8). Another US study of teens and young adults in Southern California found a 46% lower rate of gonorrhea, but not chlamydia, among recipients of MenB-4C compared with matched counterparts who had received MenACWY (9). A study from South Australia found that two doses of MenB-4C vaccination provided an effectiveness of 32.7% against gonorrhea compared to a control of chlamydia infections (10). The modest effectiveness of MenB-4C in protecting against gonococcal infections suggests that shared antigens between *N. meningitidis* and *N. gonorrhoeae* elicit an antibody (Ab) response and provide cross-reactivity. The molecular basis for this protection remains unclear.

**Figure 1:**
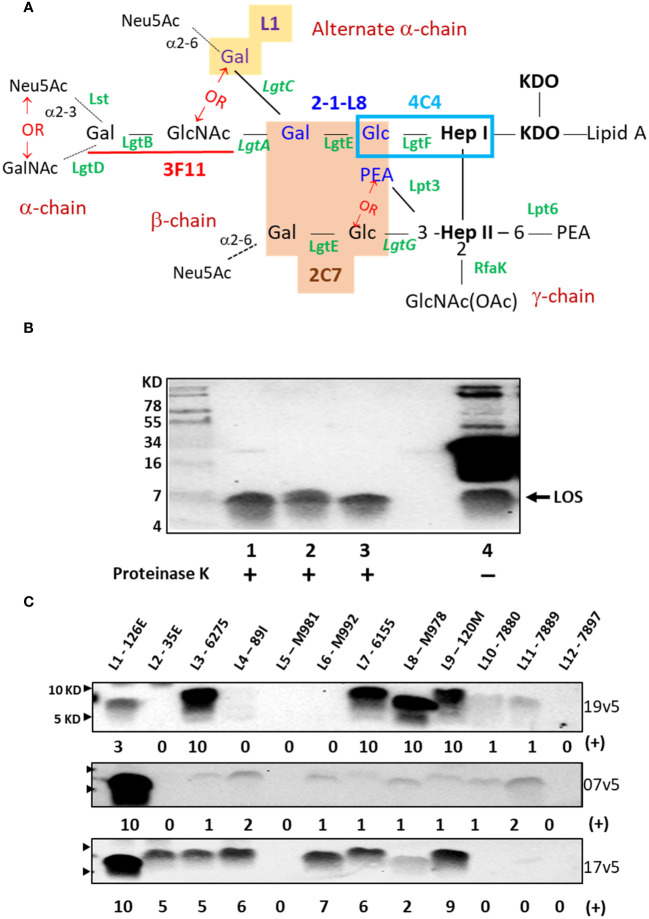
*Neisseria meningitidis* and *Neisseria gonorrhoeae* LOS assembly and structures that are recognized by IgG antibodies elicited MenB-4C immunization. **(A)** LOS structures of *N. meningitidis* and *N. gonorrhoeae* and epitope recognition by mAbs are shown. The presence of lactose (Gal-Glc) on both HepI and HepII simultaneously is required for mAb 2C7 (orange-shaded structure) (12). The mAb 2-1-L8 requires the lactose-HepI in conjunction with a 3-PEA-HepII (blue font) (13). Thus, the binding of 2C7 and 2-1-L8 occurs exclusively. The mAb 4C4 detects lactose-HepI (L8) (14) and maltose-HepI (L11) (15), pointing to a minimal structural requirement of Glc-HepI. The mAb 3F11 binds to the terminal lactosamine of the LNT structure (underlined in red); extension of the terminal Gal with either GalNAc or Neu5Ac abolishes 3F11 binding (16). The mAb L1 binds to the Gal-Gal-Glc-HepI structure (yellow shade) (17). The phase-variable LOS biosynthesis enzymes are italicized. **(B)** Western blot of meningococcal and gonococcal OMVs after PK digestion and an equivalent undigested OMV of *N. gonorrhoeae* strain 1291 was probed with post-MenB-4C sera 19v5. **(C)** Western blots of 12 defined LOS immunotypes, L1-L12, probed with post-MenB-4C human sera. Signal intensity variations within a single blot were analyzed by Image J and compared by densitometry with normalization to the strongest signal in the blot (L3 for 19v5 and L1 for 7v5 and 17v5, respectively), set as a value of 10 in a 0-10 scale. The positions of 10 KD and 5 KD prestained protein molecular weight markers are marked by arrows on the left.

The MenB-4C vaccine is composed of OMVs from the same serogroup B strain NZ98/254 used in MeNZB, three recombinant major antigens (NHBA, FHbp, and NadA), and two minor antigens that are presented as fusion proteins with NHBA (NHBA-GNA1030) and FHbp (GNA2091-FHbp). Outer membrane protein (OMP) PorA subtype P1.7-2,4 is the major protein in OMVs. Among the recombinant protein antigens in MenB-4C, *N. gonorrhoeae* does not have NadA but encodes NHBA and FHbp orthologs. PorA, the major *N. meningitidis* OMP, is also absent in *N. gonorrhoeae*. Furthermore, the gonococcal FHbp lacks a signal peptide and is not surface-exposed (11). Therefore, the recombinant *N. meningitidis* NHBA, which is ~70% conserved in *N. gonorrhoeae*, and other minor OMPs shared between *N. meningitidis* and *N. gonorrhoeae* are the potential cross-reactive protein antigens in MenB-4C. Lipooligosaccharide (LOS/endotoxin) (**Figure 1A**), the major component of the outer membrane, is readily accessible as a target of adaptive immunity and provides adjuvant activity as an additional benefit for the vaccine. Previous studies have shown that the sera of MenB-4C (18), MeNZB, and MenBVac (19) contain antibodies to *N. meningitidis* and *N. gonorrhoeae* LOS. In this study, we provide a further understanding of the LOS structures (**Figure 1A**) recognized by human serum IgG antibodies elicited by the MenB-4C vaccine and show that LOS-specific antibodies are bactericidal to *N. gonorrhoeae*. The work indicates approaches to next-generation gonococcal vaccine design.

## 2 Materials and methods

### 2.1 Bacterial isolates and growth conditions

The bacterial strains used in this study are listed in **Supplementary Table S1**. *Neisseria* were cultured on GC-base agar containing 0.4% glucose and 0.68 mM Fe(NO_3_)_3_ at 37°C and 5% CO_2_ or in GC broth with the same supplements and 0.043% NaHCO_3_ as the CO_2_ source at 37°C.

### 2.2 Outer membrane vesicle preparation

Naturally secreted OMVs of *N. meningitidis* and *N. gonorrhoeae* are isolated from overnight GC broth cultures. Cultures were treated with 0.05% sodium azide and incubated at 60°C for 30 min to kill bacteria, and then bacteria were removed by centrifugation at 5,000×*g* for 15 min. The supernatant was cleared by PES filter (0.45 μm), concentrated with Centricon Plus 70 filtration units with a 100-kDa cut-off (Thermo Fisher Scientific, Waltham, MA) at 3,500×*g*, and then OMV collected by ultracentrifugation (100,000×*g*, 80 min, 4°C). The resulting pellets containing OMVs were washed three times with PBS and then solubilized in PBS containing 0.2% SDS. The protein concentration was measured using a BCA protein assay (Thermo Fisher Scientific, Waltham, MA).

### 2.3 Human sera

The anonymized-residual, pre- and postvaccine human sera used in this study were obtained from a vaccine clinical trial of MenB-4C in laboratory workers in the Public Health Laboratory, Manchester, UK (20); www.clinicaltrials.gov identifier: NCT00962624. Enrollment was open to staff aged 18-65 years. MenB-4C was administered at 0 month, 2 months, and 6 months in the nondominant arm and ACWY-CRM concomitantly at 0 months in the dominant arm. Samples used in this study were obtained at 0 months (visit 1, v1) and 1 month after the third dose, (visit 5, v5).

### 2.4 LOS purification

L3 and L5 LOS from the respective immunotyping strains 6275 and M981 were isolated following the previously described phenol-chloroformpetroleum ether extraction procedure (21).

### 2.5 Western blots

To study vaccine-induced anti-LOS IgG antibodies, proteins in purified meningococcal and gonococcal OMV preparations were digested by proteinase K (PK) prior to probing by Western blots with human sera obtained pre- and postimmunization with MenB-4C. LOS expression and LOS structures were confirmed with a collection of five monoclonal antibodies (mAbs), 3F11, L1, 2C7, 4C4, and 2-1-L8, directed at meningococcal and gonococcal LOS. Aliquots of 15 μg OMVs were incubated at 60°C overnight with 100 μg of PK in the presence of 1.5% SDS in a total volume of 30 μL. After the digestion, 30 μL of 2× Tricine sample buffer was added. Aliquots containing 1 μg of OMV protein were resolved by 16.5% Tris-Tricine gel (Bio-Rad, Hercules, CA) and then transferred to the PVDF membrane. Precision Plus Protein™ Dual Xtra Prestained Protein Standards (Bio-Rad, Hercules, CA) was used as the molecular weight marker. The membrane was blocked with Block Buffer of 5% nonfat dry milk (NFDM) in 1 × TBS for 1 h at RT and then probed with MenB-4C serum 19v5 (1:5,000) in 2.5% NFDM-0.1% Tween-1X TBS overnight at 4°C. After 3-min × 5-min washes with 1× TBST + 0.1% Tween 20, the membrane was incubated with horse radish peroxidase (HRP)-conjugated secondary Ab IgG-HRP at 1:10,000 dilution for 1 h at room temperature, followed by three additional 15-min washes. The signals were developed with West Pico Plus (Thermo Fisher Scientific, Waltham, MA) and recorded with an I Bright 20 scanner. When working with whole cell lysates, overnight plate-grown bacteria at 0.1 OD_550_ was resuspended in 10 μL of 1.5% SDS in TE buffer and 5 μL of 100 μg/mL proteinase K. The mixtures were incubated at 60°C overnight. Aliquots of 7.5 μL of the digested mixture were mixed with 7.5 μL of 2× Tricine sample buffer premixed with 5% β-mercaptoethanol and boiled for 10 min before loading. Signal intensity variations within a single blot were compared by densitometry with normalization to the strongest signal in the blot (set as a value of 10 on a 0-10 scale). The mouse mAbs as hybridoma cell supernatants were used at a 1:100 dilution together with anti-mouse IgM-HRP at 1:1,000 for 3F11 and anti-mouse IgG-HRP at 1:3,000 for all other mAbs. The monoclonal antibodies developed by P. A Rice (L1, 2C7, and 2-1-L8) and developed by M. Apicella (3F11 and 4C4) were obtained from the Developmental Studies Hybridoma Bank, created by the NICHD of the NIH and maintained at the University of Iowa. We next examined whether the structure of lactose-HepI + phosphorylated HepII without 3-Glc competed away the post-MenB-4C signals to LOS immunotypes with extended α-chains. The 19v5 serum was preincubated with cells of the L8 prototype strain M978 to remove Abs binding to the truncated LOS structures or with the L2 prototype strain 35E to titrate away Abs targeting an intact α-chain (1 h incubation with cells at 0.2 OD_550_ per mL at RT, and then cells were removed by centrifugation). A mock incubation without cells was also performed as a control. The absorbed sera were subsequently used to probe five purified 19v5-positive LOS immunotypes (L1, L3, L7, L8, and L9).

### 2.6 Genome survey

The PubMLST database was searched on 16 January 2024. The query was limited to isolates with a total WGS length of at least 2 Mbp, which resulted in 19,555 *N. gonorrhoeae* genome records. The “Gene Presence” analysis tool available as a plug-in at PubMLST was used to examine six loci: neis1986 (*lpt3*), neis2010, neis2011 (*lgtG*), neis2012 (*lpt6*), neis2014 (*gmhB*), and neis2015 (*nlaB*). The default parameters of minimal identity at 70%, minimal alignment at 50%, and a BLASTN word size of 20 were applied.

### 2.7 Serum bactericidal assays with human complement

SBA protocol was as described (22). A commercial IgG- and IgM-depleted normal human serum pool (Pel-Freez, Rogers, AR) is used as the complement source at 10% and assayed with serum collected after MenB-4C vaccination, of which the complement has been heat inactivated at 56°C for 30 min. Strains grown in supplemented GC broth to mid-log phase of OD_550_ at ~ 0.4 - 0.7 were adjusted to 0.12 for *N. meningitidis* and 0.15 for *N. gonorrhoeae* with clear RPMI Media and further diluted 1:2,500 in RPMI. A 20-μL aliquot of bacterial suspension was added to a mixture (60 μL) of pooled complement source (8 μL) and diluted test sera in a 96-well plate to start the assay. After 1-h incubation at 37°C, 40-μL aliquots were diluted with 160 μL of RPMI, and 25 μL of the dilutions were spotted onto GC agar plates using the tilted plating method. Viable counts were obtained after overnight incubation. When needed, 50 ng of purified L3 LOS or L5 LOS, which were isolated from the immunotyping strains 6275 and M981, respectively, was added and incubated at 37°C for 30 min prior to the addition of bacteria. The wells with 10% Pel-Freez sera only, which has no bactericidal activity up to 40%, were set at 100% for normalization.

### 2.8 Statistical analysis

A one-way ANOVA with an uncorrected Fishers least significant difference test or two-tailed unpaired Students *t*-tests were performed to compare serum bactericidal activities. The *p*-values less than 0.05 (^*^) and 0.001 (^***^) were considered statistically significant.

## 3 Results

### 3.1 MenB-4C immunization elicits *N. meningitidis* and *N. gonorrhoeae* LOS-specific IgG antibodies

Initial data demonstrated that post-MenB-4C sera recognized both meningococcal and gonococcal LOS species. As shown in **Figure 1B**, proteinase K (PK)-digested OMVs probed with a post-MenB-4C serum (19v5) showed LOS reactive bands for two *N. gonorrhoeae* strains, 1291 and CNG20, as well as the *N. meningitidis* strain H44/76 used to generate the Norwegian serogroup B OMV vaccine, MenBVac (23). Such signals constitute a fraction of the post-MenB-4C IgG antibodies that recognize meningococcal and gonococcal protein antigens, shown by multiple bands in undigested gonococcal 1291 OMVs (**Figure 1B**, lane 4). Densitometry analysis estimated that antibodies against LOS accounted for ~10% of the total IgG response to OMV antigens in gonococcal strain 1291. No LOS signal was detected when Western blots were probed with the preimmune 19v1 sera from the same individual, indicating that MenB-4C immunization elicited IgG antibodies to *N. meningitidis* and *N. gonorrhoeae* LOS.

*N. meningitidis* expresses twelve LOS immunotypes, L1 - L12, which are defined by structural differences (**Table 1**) (29, 38). PK digests of the L1 - L12 LOS immunotyping strains were probed with three post-MenB-4C sera (**Figure 1C**). Each serum was quite distinct in the recognition of LOS immunotypes in addition to both L1 and L3 LOSs present in the strain NZ98/254 OMV component of MenB-4C. With serum 19v5, LOS of immunotypes L3, L7, L8, and L9 reacted intensely, and immunotypes L10 and L11 reacted weakly, but immunotypes L2, L4, L5, L6, and L12 did not react. The 7v5 serum recognized L1 LOS, with faint signals detected for immunotypes L3, L4, and L6 - L11 (~3% - 12% of L1) and no signal for L2, L5, and L12 LOS. Lastly, serum 17v5 recognized most LOS immunotypes, intensely with L1 LOS and immunotypes L2, L3, L4, L6, L7, and L9 (~50% - 90% of L1), but L8 reactivity was diminished (~20% of L1), and no signal was detected for L5 and L10 - L12 LOS. These data indicated that LOS-specific Ab populations generated are different in individuals vaccinated with MenB-4C.

**Table 1:** Structures of *N. meningitidis* LOS immunotypes and prototype strains.

Immunotype (strain)	α-Chain (HepI)	β-Chain (HepII)^f^		γ-Chain (HepII)	Reference
		3-	6-	2-	
L1 (126E)	Gal_α1→4_Gal_β1→4_Glc_β1→4_ Neu5Ac_α2→6_Gal_α1→4_Gal_β1→4_Glc_β1→4_	PEA^a^	H	GlcNAc(OAc)	(17)
L2 (35E)	Neu5Ac_α2→3_Gal_β1→4_GlcNAc_β1→3_Gal_β1→4_Glc_β1→4_	Glc	PEA	GlcNAc(OAc)	(24)
L3 (6275)	Neu5Ac_α2→3_Gal_β1→4_GlcNAc_β1→3_Gal_β1→4_Glc_β1→4_	PEA^a^	H	GlcNAc	(25)
L4 (89I)	Neu5Ac_α2→3_Gal_β1→4_GlcNAc_β1→3_Gal_β1→4_Glc_β1→4_	H	PEA	GlcNAc(OAc)	(26)
L5 (M981)	Gal_β1→4_GlcNAc_β1→3_Gal_β1→4_Glc_β1→4_Glc_β1→4_	Glc	H	GlcNAc(OAc)	(27)
L6 (M992)	GlcNAc_β1→3_Gal_β1→4_Glc_β1→4_	H	PEA^c^	GlcNAc(OAc)	(26, 28)
L7 (6155)	Gal_β1→4_GlcNAc_β1→3_Gal_β1→4_Glc_β1→4_	PEA^a^	H	GlcNAc	(26)
L8 (M978)	Gal_β1→4_Glc_β1→4_	PEA^a^	H	GlcNAc	(29)
L9 (120M)	Gal_β1→4_GlcNAc_β1→3_Gal_β1→4_Glc_β1→4_	PEA^a^	H/PEA^d^	GlcNAc(OAc)	(30)
L10 (7880)	Gal_β1→4_Glc_β1→4_Glc_β1→4_-	PEA^a^	nd	GlcNAc(OAc)^e^	(31)
L11 (7889)	Glc_β1→4_Glc_β1→4_-	PEA^a^	PEA	GlcNAc(OAc)	(32)
L12 (7897)^b^	nd	PEA^a^	nd	GlcNAc(OAc)^e^	(33)

a The LOS of these immunotyping *N. meningitidis* strains reacts positively with mAb B5, which specifically recognizes inner core structures that contain a PEA attached at the 3-position on of HepII, as reported by Mackinnon et al. (34).

b No structural characterization has been reported for L12 LOS. However, L12 LOS migrates faster than L8 and L11 LOS on silver-stained SDS-PAGE (33), thus L12 LOS is a lower MW species, likely an extensively truncated LOS species, and does not react with post-MenB-4C sera.

c L6 LOS was reported to have a 7-PEA and no 6-PEA on HepII (26); a subsequent NMR study demonstrated that the PEA is located at the six-position (28).

d The presence of a single 6-PEA of HepII was reported for the L9 strain Z2491 (lgtG-, lpt3-) (30). However, the immunotyping strain 120 M encodes lpt3 and is mAb B5 positive (i.e., carrying 3-PEA).

e The gene, lot3, encoding the O-acetyltransferase of 2-GlcNAc on HepII is identified in all immunotyping strains. Thus, the GlcNAc of L10 and L12 immunotypes are predicted to be O-acetylated (35); however, there is no biochemical structure determination.

f Glycine at the 7-position of HepII has been reported to be present in the L2 strain NMB (36) as well as the L3 and L4 immunotypes (37). The attachment of glycine to the inner cores of L1 and L5L7 immunotypes has not been investigated.

nd, not determined.

The lactoneotetrose (LNT, Gal-GlcNAc-Gal-Glc) α-chain is present in L2, L3, L4, L7, and L9 LOS structures (**Table 1**), but immunotypes L2 and L4 LOSs did not react with serum 19v5 (**Figure 1C**). Serum 19v5 recognized L1 LOS with the alternate α-chain (Gal-Gal-Glc-HepI) and the truncated L8 LOS (Gal-Glc-HepI). All 19v5-reactive immunotypes also carry a phosphoethanolamine (PEA) at the 3-position of HepII (L1, L3, L7 - L11), while the immunotypes not recognized by 19v5 have either 3-Glc (L2, L5) or 3-H (L4, L6) at HepII (**Table 1**). This profile suggested that the majority of LOS antibodies in the serum 19v5 did not require an intact α-chain (LNT or L1) but were directed at structures of a truncated α-chain together with phosphorylated HepII shared by meningococcal and gonococcal LOS. In addition, absorbing serum 19v5 with the prototype L8 strain M978, but not the L2 strain 35E, markedly reduced the signals of L1, L3, L7, and L9 that have intact LOS α-chain (**Supplementary Figure S1**). These data further support the finding that the dominant LOS-specific Abs in 19v5 serum target structures of phosphorylated HepII with a truncated alpha-chain (i.e., L8). However, we cannot rule out the presence of a small fraction of Abs in 19v5 serum that is directed against the longer α-chains.

In comparison, the immunodominant LOS-specific antibodies in serum 17v5 recognized L1 LOS and a higher molecular weight (MW) band, representing an extended LNT α-chain. All immunotypes with a complete LNT were reactive with serum 17v5. L6 LOS missing the terminal galactose (29) remained 17v5 reactive, but further truncation of the α-chain to the L8 structure significantly diminished 17v5 recognition. The HepII inner core decoration at the 3-position was not critical for serum 17v5 reactivity, as either 3-Glc, 3-PEA, or 3-H were found in various 17v5-reactive immunotypes. Thus, the α-chain structures with a minimum of 3 sugars, GlcNAc-Gal-Glc-HepI (i.e., L2, L3, L4, L6, L7, and L9) or Gal-Gal-Glc-HepI (i.e., L1), were the targets for LOS recognition by serum 17v5.

**Figure 2:**
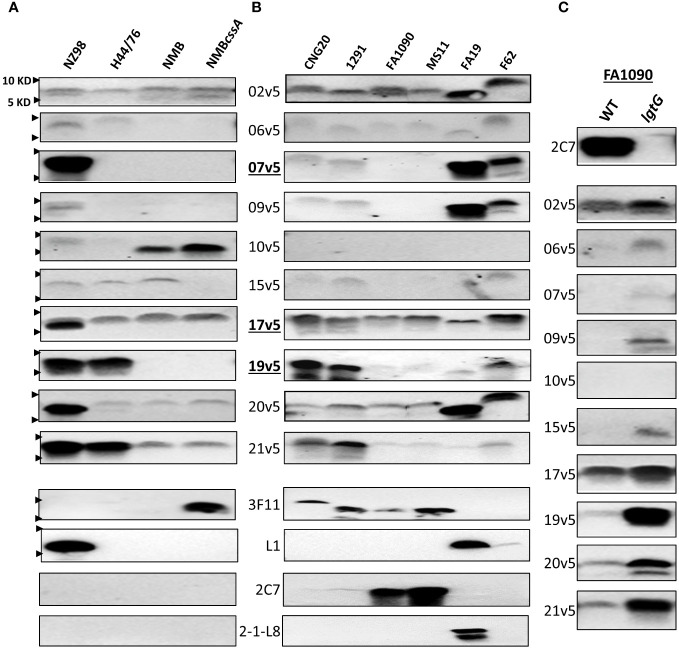
Immunodominant LOS-specific IgG antibody responses to *N. meningitidis* and *N. gonorrhoeae* differ among MenB-4C-vaccinated individuals. **(A)** LOS of representative *N. meningitidis* strains probed with 10 post MenB-4C sera and four relevant LOS-specific mAbs (3F11, L1, 2C7, and 2-1-L8). PK-digested whole cell lysates were resolved, and Western blots performed as described in the procedures. The positions of 10 KD and 5 KD prestained protein molecular weight markers are marked by arrows on the left. **(B)** Five *N. gonorrhoeae* reference strains (see **Supplementary Tables S2** for structures) and one 2015 clinical isolate CNG20 were analyzed as in **(A)**. **(C)** The 2C7-positive gonococcal strain FA1090 and its 2C7-negative *lgtG* mutant were probed with 10 post-MenB-4C sera. Loss of mAb 2C7 epitope in FA1090 enhanced *N. gonorrhoeae* recognition by most MenB-4C sera.

The L5, L10, and L11 immunotypes with a maltose (Glc-Glc) instead of a lactose (Gal-Glc) linkage to HepI (**Table 1**) did not react or react weakly with each MenB-4C sera. Among the L5, L10, and L11 immunotypes, L5 has a 3-Glc-HepII moiety and no PEA. L5 LOS was not recognized by the three post-MenB-4C sera, while L10 and L11 with a 3-PEA-HepII reacted weakly with 19v5 and 7v5 sera (~10% of the most reactive immunotype). Sialylation of the terminal Gal of the LNT was not required for Ab binding since both the sialylated (L3) and nonsialylated forms (L7, L9) of the LNT moieties reacted with each of the three sera in Western blots. Consistently, the nonsialylated gonococcal LOS structures were also recognized by post-MenB-4C sera (**Figures 1B**, **2B**, below), further indicating that LOS sialylation did not significantly interfere with Ab binding.

### 3.2 MenB-4C elicited LOS-specific IgG antibodies for different LOS structures of *N. meningitidis*

With the evidence that IgG responses to LOS differed among individuals after MenB-4C vaccination, we studied ten post MenB-4C sera using additional meningococcal and gonococcal strains (**Figure 2**). For the meningococcal panel, the MenB-4C vaccine strain NZ98/254, strain H44/76, strain NMB and the NMB*cssA* mutant expressing nonsialylated LOS (39) were included. As noted, strain H44/76 was used for MenBVac, the Norwegian serogroup B OMV vaccine (23), and had been reported to express L3 and nonsialylated (L7) or truncated (L8) LOS structures. The group B strain NMB, producing predominantly L2 and L4 LOS (40), has been used extensively in the study of meningococcal LOS assembly with detailed structural characterizations performed for multiple mutants in the LOS biosynthesis pathway (36, 41–43). Data with the mAb 3F11 (**Figure 2A**), which only recognizes nonsialylated LNT (44), indicated that strains NZ98/254, H44/76 and NMB were fully sialylated. Probing with mAb L1, which specifically recognizes the alternate L1 α-chain (Gal-Gal-Glc-HepI, **Figure 1A**), confirmed that only the vaccine strain NZ98/254 produced L1 LOS (**Figure 2A**). No signals were detected when probed with mAb 2C7 that targets 3-lactose-HepII/lactose HepI, or with mAb 2-1-L8 that recognizes a truncated α-chain (Gal-Glc-HepI) with a 3-PEA-HepII (**Figure 1A**). Recognition by mAb 2-1-L8 is known to be masked by an intact α-chain (45).

All 10 post-MenB-4C sera reacted, with different intensities, to the LOS of NZ98/254 (**Figure 2A**). Half of the post-MenB-4C sera (7v5, 17v5, 19v5, 20v5, and 21v5) showed strong anti-LOS signals to NZ98/254 LOS. Variability in the recognition of other meningococcal LOSs by post-MenB-4C sera was also seen. For example, serum 10v5, but not most other sera, recognized a lower MW structure of NMB and NMB*cssA* (**Figure 2A**). In contrast, serum 19v5 showed strong reactivity to the LOSs of NZ98/254 and H44/76, but not to the LOS of NMB and NMB*cssA*. Strain H44/76 expressing L3 (but not L1) demonstrated reactivity with sera 17v5, 19v5, and 21v5, but weakly (2v5, 6v5, and 15v5) or not at all with the other sera. Serum 20v5 was weakly reactive with a high MW LOS species of H44/76, NMB, and NMB*cssA*. The 17v5 serum recognized two LOS bands of strain NZ98/254, one specific for NZ98/254 not seen in H44/76 and one higher LOS band found in all tested *N. meningitidis* strains. The 2v5 serum showed relatively equal intensities among all four *N. meningitidis* strains. In contrast, the serum 9v5 only reacted with LOS of the vaccine strain NZ98/254.

The reactivity profiles of three post-MenB-4C sera toward the 12 N*. meningitidis* immunotypes (**Figure 1C**) implied that LOS sialylation did not interfere with recognition. The impact of LOS sialylation on Ab recognition was further addressed with the additional MenB-4C sera. MenB-4C sera (**Figure 2A**) recognized the sialylated NZ98/254, H44/76, and NMB LOSs as well as the nonsialylated NMB*cssA* LOS, again indicating that LNT sialylation was not critical for recognition by LOS antibodies in post MenB-4C sera. One exception was sera 15v5, which was reactive only for sialylated LOS samples (**Figure 2A**). In summary, the Western blot data on the meningococcal LOS panel confirmed the heterogeneity of LOS species expressed by meningococci and that LOS antibodies of individual post-MenB-4C sera recognized different LOS structures.

### 3.3 MenB-4C elicited LOS-specific IgG antibodies for different LOS structures of *N. gonorrhoeae*

For the *N. gonorrhoeae* panel, we studied the LOS of six *N. gonorrhoeae* strains: CNG20 (a 2015 N*. gonorrhoeae* clinical isolate), 1291, FA1090, MS11, FA19, and F62. The LOS structures of 1291, FA19, MS11 and F62 have been previously reported (**Supplementary Table S2**), while those of FA1090 and CNG20 were predicted based on mAb reactivity (**Figure 2B**). Four (CNG20, 1291, FA1090, and MS11) of the six *N. gonorrhoeae* strains were 3F11-positive, indicating the presence of nonsialylated LNT α-chain (**Figure 2B**). The published structure for 1291 (**Supplementary Table S2**) (46, 47) is consistent with L7 LOS (nonsialylated L3). Two of these four *N. gonorrhoeae* strains (FA1090 and MS11, **Figure 2B**) also showed strong mAb 2C7 signals. Strain FA19 had both L1 and 2-1-L8 signals but was 3F11 negative, indicating FA19 produced LOSs with truncated and alternate α-chains. F62, which has a GalNAc substitution on the terminal galactose of LNT (48) was weakly positive for mAb L1, but negative for 3F11, 2C7, and 2-1-L8.

Three of ten post-MenB-4C sera - 2v5, 17v5, and 20v5 - demonstrated broad cross-reactivity to gonococcal LOS (**Figure 2B**). Four other post-MenB-4C sera (7v5, 9v5, 19v5, 21v5) were discriminatory for *N. gonorrhoeae* WT LOS structures (**Supplementary Table S2**), two others reacted weakly with gonococcal LOS (6v5 and 15v5) and one did not recognize *N. gonorrhoeae* LOS (10v5). The three post-MenB-4C sera that showed strong signals to LOS of the *N. meningitidis* strain H44/76 (17v5, 19v5, and 21v5, **Figure 2A**), also reacted strongly with the LOS of both *N. gonorrhoeae* CNG20 and 1291. Sera 7v5 and 20v5, which were only strongly reactive with L1 LOS in NZ98/254 (**Figure 2A**), displayed weak signals to LOSs of CNG20 and 1291. The LOS of CNG20 had a higher MW than the L7 LOS of 1291, possibly due to additional glycan substitutions. LOS structures expressed by strain FA1090 and MS11 were recognized by fewer sera (2v5, 17v5, and 20v5) than other *N. gonorrhoeae* strains. FA19 LOS was recognized by most post-MenB-4C sera and strongly with 4 sera (2v5, 7v5, 9v5, and 20v5) at a lower MW than those of other *N. gonorrhoeae* strains. Most post-MenB-4C sera also recognized LOS expressed by *N. gonorrhoeae* F62, identified as a higher MW band, consistent with the reported terminal GalNAc addition on the α-chain (**Supplementary Table S2**). Loss of the 2C7 epitope in strain FA1090*lgtG*, created by an *lgtG* mutation that eliminates the lactose addition to the 3-position of HepII (**Figure 2C**, top), enhanced the recognition by most of the post-MenB-4C sera compared to the parental FA1090 LOS (**Figure 2C**). Of ten sera tested with the FA1090*lgtG* mutant, the 10v5 serum, which overall does not recognize *N. gonorrhoeae* LOS (**Figure 2B**), was the exception. In summary, the Western blot data on the gonococcal LOS panel confirmed that LOS antibodies of individual post-MenB-4C sera recognized distinct gonococcal LOS structures, and the removal of the 3-lactose-HepII enhanced recognition.

### 3.4 Meningococcal and gonococcal LOS structures recognized by LOS-specific IgG antibodies

Based on these initial studies, antibodies in post-MenB-4C sera detected different LOS structural epitopes. To further define the *N. meningitidis* and *N. gonorrhoeae* LOS epitope(s) recognized by IgG antibodies elicited by MenB-4C vaccination, a collection of LOS biosynthesis mutants of two *N. meningitidis* and two *N. gonorrhoeae* strains with LOS structures defined by biochemical studies or mAb reactivities were further probed in detail with three post-MenB-4C sera. Stains used included the *N. meningitidis* strain NMB expressing both L2 and L4 LOS structures (36, 41–43), the vaccine strain NZ98/254 producing both L1 and L3 LOSs, and two gonococcal strains, FA19 and F62, which have well-characterized LOS structures (46, 48). The mutations investigated in these strains included *lgtG* (49), *lpt3*, *lpt6*, *lpt3/lpt6* (34, 36), *lpt6/lgtG*, *lgtA* (50), *lgtA*/*lgtC*, *lgtF* (51), *rfaK* (21, 36, 43), *galE* (41), *galE/lgtG*, and *cssA* (**Figure 1A**).

#### 3.4.1 *N. meningitidis* strain NMB

**Figure 3:**
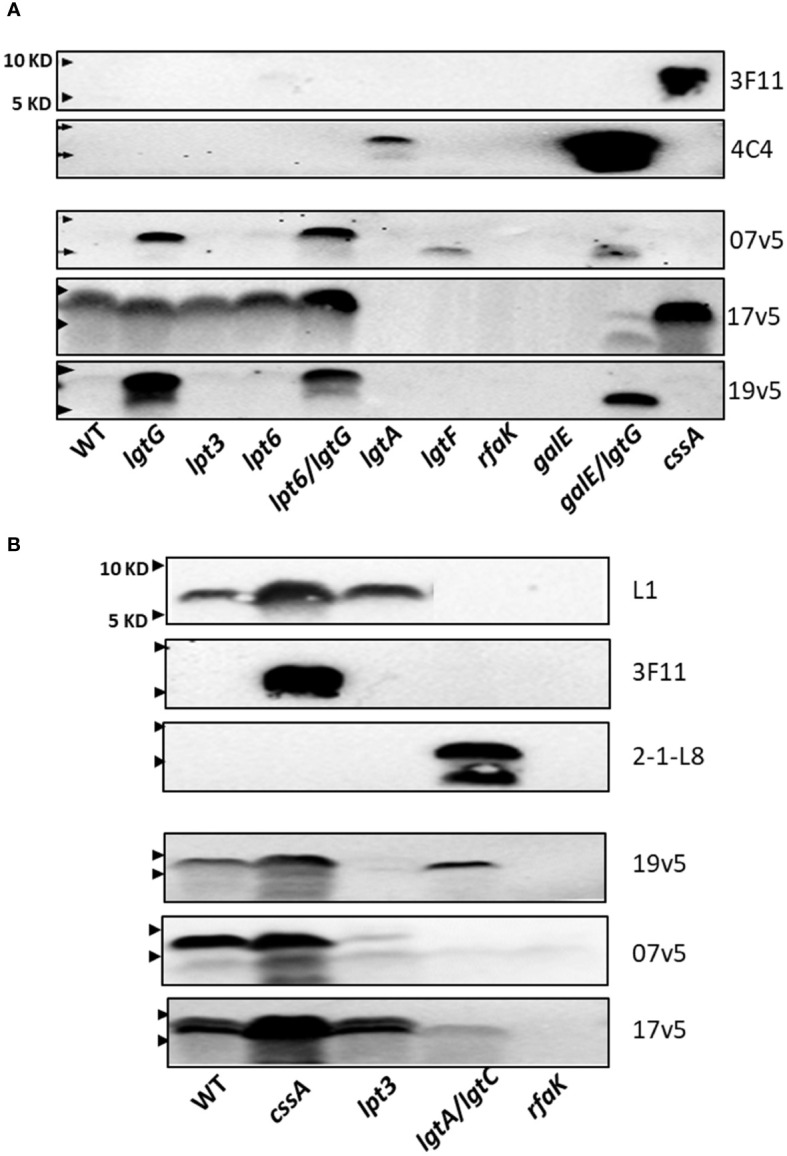
Recognition of *N. meningitidis* LOS structures by post-MenB-4C sera. **(A)** Western blots of *N. meningitidis* parental NMB and mutants, as probed by three post MenB-4C sera (7v5, 17v5, and 19v5) and two mAbs 3F11 and 4C4. The loss of glucose on HepII allowed recognition of *N. meningitidis* NMB LOS; all three post-MenB-4C sera recognized LOS in the genetic background of *lgt*G mutations. **(B)** LOS Western blots of the vaccine strain NZ98/254 and mutants. Both sialylated and unsialylated LOS were recognized by all three sera, but the removal of 3-PEA by the *lpt*3 mutation eliminated recognition by sera 19v5 and 7v5. The positions of 10 KD and 5 KD prestained protein molecular weight markers are marked by arrows on the left.

The LOS structures of strain NMB and mutant are shown in **Supplementary Table S3**. There was no reactivity with mAbs L1 and 2-1-L8. The NMB*lgtA* mutant was weakly reactive with mAb 4C4 (**Figure 3A**) and not reactive with 2-1-L8, indicating that the NMB*lgtA* mutant had no 3-PEA-HepII and instead was mostly substituted with 3-Glc. The NMB*galE/lgtG* mutant was strongly reactive with mAb 4C4, but the NMB*galE* mutant (41, 52) was 4C4 negative. The mAb 4C4 recognizes both Gal-Glc-HepI and Glc-HepI structures (53) as well as Glc-Glc-HepI of the L11 LOS (15), thus the 3-Glc-HepII moiety of the NMB*galE* mutant likely prevented 4C4 binding. When the NMB panel was probed with 3F11, only the nonsialylated NMB*cssA* mutant was reactive, indicating that the LNT α-chains of WT and the inner core mutants (NMB*lgtG*, *lpt3*, *lpt6*, and *lpt6/lgtG*) were sialylated.

These defined NMB LOS structures were probed with three post-MenB-4C sera (**Figure 3A**). LOS species of both the WT and the nonsialylated *cssA* mutant were not recognized by sera 19v5 and 7v5 but were reactive with 17v5, confirming the initial results shown in **Figure 2A**. The LOS of the NMB*lgtG* mutant, also devoid of 3-PEA addition to HepII (54), was now recognized by sera 19v5 and 7v5, but the *lgtG* mutation did not alter reactivity for serum 17v5 (**Figure 3A**, lane 2). The recognition of the WT*lgtG* was extended to the LOSs of the *lpt6/lgtG* and *galE/lgtG* mutants (lanes 5 vs. 4 and 10 vs. 9). A shared feature among WT, *lpt6*, and *galE* mutants is the 3-Glc-HepII moiety, a defining feature for the L2 immunotype. As a structural characterization of NMB*lpt3* LOS has shown complete glycosylation at the 3-position of HepII (36) (**Supplementary Table S3**), the lack of 19v5 and 7v5 reactivities with this mutant is also correlated with blocking by 3-Glc-HepII. In addition, the NMB*lgtA* mutant, which presumably retained the 3-Glc-HepII, was also not reactive for sera 19v5 and 7v5. Thus, the presence of 3-Glc on HepII interfered with Ab recognition by 19v5 and 7v5, regardless of an intact (WT and *lpt6*) or a truncated α-chain (*lgtA* and *galE*). These data were consistent with the observation of the FA1090*lgtG* LOS being significantly more reactive than the parental FA1090 LOS when probed with most of the post-MenB-4C sera (**Figure 2C**). The NMB*lgtG* mutant with a 6-PEA and the NMB*lpt6/lgtG* mutant predicted to have a 3-PEA on HepII were both reactive with sera 19v5 and 7v5, suggesting that, while PEA on Hep II was critical, the linkage of PEA (either 3-PEA or 6-PEA) was not important for LOS antibodies in these two post-MenB-4C sera to recognize the LOS species.

The NMB*lgtF* mutant was recognized by 7v5, but not by 19v5. Another deeply truncated LOS, the NMB*rfaK* mutant, was not reactive with either sera. The LOS of NMB*lgtF* has no sugar added to either α-chain or β-chain but retains the 2-GlcNAc of the γ-chain (41), while the NMB*rfaK* mutant has further lost γ-chain glycosylation (36, 43) (**Figure 1A**; **Supplementary Table S3**). The importance of 2-GlcNAc-HepII in triggering Ab responses has been shown by synthetic oligosaccharides (55). The difference between *lgtF* and *rfaK* LOSs indicated that, when HepI is not glycosylated, the 2-GlcNAc on HepII may be sufficient for 7v5 Ab binding but insufficient for 19v5. These results from the NMB genetic background indicated that post-MenB-4C sera 19v5 and 7v5 have no requirement for an intact or sialylated LNT α-chain, and LOS-specific antibodies likely recognized LOS structure(s) with at least a Glc extension on HepI (19v5) and a 2-GlcNAc on HepII (7v5). The presence of PEA at either 3- or 6-positions of HepII was not as critical, but the 3-Glc-HepII completely blocked the recognition by sera 19v5 and 7v5. In contrast, the major LOS antibodies in sera 17v5 recognize structures that have an intact LNT (or L1) α-chain. Mutations causing truncation in the α-chain (*lgtA*, *lgtF*, *rfaK*, and *galE*) eliminated recognition by the 17v5 serum. Serum 17v5 has a reduced signal for truncated α-chain structures (**Figure 1C**), explaining the weak signal for the low MW LOS of the NMB*galE/lgtG* mutant (**Figure 3A**). Unlike 7v5 and 19v5, the 3-Glc-HepII did not block binding of 17v5 antibodies, as shown by signals for the WT, the *lpt3*, and the *lpt6* mutants.

#### 3.4.2 *N. meningitidis* strain NZ98/254

Mutations in *cssA*, *lpt3*, *lgtA/lgtC*, and *rfaK* were examined in strain NZ98/254. Strain NZ98/254 does not encode the *lgtG*-*lpt6* genetic island critical for LOS inner core modifications and expresses L1 and L3 LOS structures that differ in α-chain compositions, but both structures have a 3-PEA on Hep II (**Table 1**). Terminal 2,6-linked sialylation of L1 LOS has been reported in *N. meningitidis* (17, 56), and *N. gonorrhoeae* (57). The stronger L1 signal in the *cssA* mutant implied that L1 LOSs in the WT and the *lpt3* mutant of NZ98/254 were partially sialylated. In contrast, 3F11 was only positive in the *cssA* mutant, suggesting that the LNT α-chain of L3 LOS was sialylated in WT and the *lpt3* mutant.

When probed with the three post-MenB-4C sera (**Figure 3B**), stronger signals were detected for the LOS of the NZ98/254*css*A mutant than that of the WT, implying LOS-specific antibodies in the post-MenB-4C sera have greater affinities for unsialylated LOS species. The LOS of the NZ98/254*lpt3* mutant, which has no modification on HepII (*lpt3*, *lpt6*, and *lgtG* are absent in the genome), essentially lost reactivity when probed with sera 7v5 and 19v5. Thus, a single PEA on HepII (either 3-PEA or 6-PEA) was needed to fulfill the epitope requirement for sera 7v5 and 19v5. The NZ98/254*lpt3* mutant remained positive for 17v5, as a PEA-HepII substitution was not critical. The LOS of the NZ98/254*rfaK* mutant, like the mutant in strain NMB, did not react with MenB-4C sera and all mAbs, indicating this deeply truncated structure, lacked the necessary LOS epitopes.

#### 3.4.3 *N. gonorrhoeae* strain FA19

**Figure 4:**
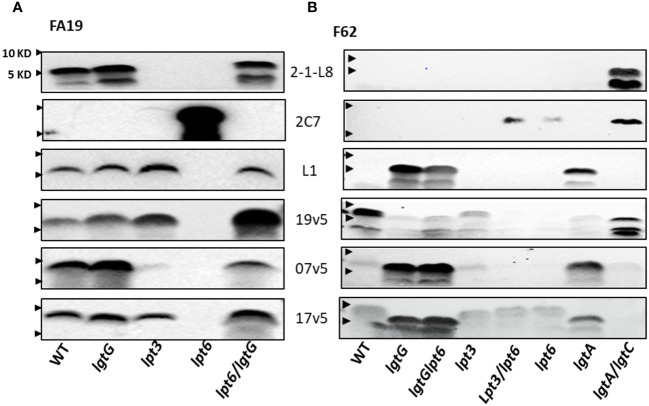
Recognition of LOS structures in *N. gonorrhoeae* strains by post-MenB-4C sera. **(A)** Western blots of strain FA19 and four mutants. Blots probed with three mAbs (2-1-L8, 2C7, and L1) and three post-MenB-4C sera are shown. **(B)** Western blots of the gonococcal strain F62 and seven mutants probed with the same mAbs and sera as in **(A)**. *Lpt*6 mutations in FA19 and F62 have strong 2C7 signals that were no longer recognized by post-MenB-4C sera. An additional *lgt*G mutation rescued reactivity. The positions of 10 KD and 5 KD prestained protein molecular weight markers are marked by arrows on the left.

The FA19 strain produces L8 LOS, as reported by Shafer et al. (58), and L1 LOS (**Figure 4A**) (46). A LNT in the α-chain was not detected with mAb 3F11 (**Figure 2B**). The 2-1-L8 signals of the WT, the FA19*lgtG* and FA19*lpt6/lgtG* mutants (**Figure 4A**), also indicated the presence of 3-PEA on HepII. The 2-1-L8 signal, as expected, disappeared in the *lpt3* mutant. We detected doublet bands in the 2-1-L8 blots of L8-positive samples (**Figures 2B**, **4A**). The 2-1-L8 mAb does not react with Glc-HepI (17). Thus, this faster migrating band may be a variant of L8 LOS with structural change(s) at the basal region (e.g., lipid A head groups). A *lpt6* mutation eliminated L1 LOS expression and converted FA19 LOS to strongly 2C7 reactive and 2-1-L8 nonreactive, indicating lactose substitution (Gal-Glc) at the 3-position of HepII. This profile indicated either that *lgtG* in the WT FA19 was phase-off but switched to phase-on in the FA19*lpt6* mutant clone or that the 6-PEA on HepII interfered with the Gal extension on 3-Glc-HepII, a phenomenon not previously described. The 2C7 signal of the FA19*lpt6* mutant predictably disappeared with the additional *lgtG* mutation (**Figure 4A**), and the FA19*lpt6/lgtG* double mutant again became mAb 2-1-L8 reactive.

Because the dominant LOS species of WT FA19 are L8 and L1 with a 3-PEA on HepII the binding of sera 7v5 and 19v5 to LOS was not affected by a *lgtG* mutation (**Figure 4A**). Binding of 19v5 was not affected by the absence of 3-PEA in FA19*lpt3* (**Figure 4A**), likely due to the presence of 6-PEA-HepII. However, serum 7v5 binding was markedly reduced due to the absence of 3-PEA on the immunodominant L1 LOS. Serum 17v5 binding to FA19 LOS was not altered by the *lpt3* or *lgtG* mutations (**Figure 4A**), consistent with the notion that 17v5 did not require a specific HepII structure. WT FA19 LOS (2C7 nonreactive) was recognized by at least half of the post-MenB-4C sera (**Figure 2B**); conversely, the 2C7-positive FA19*lpt6* LOS was not reactive, while the 2C7-negative FA19*lpt6/lgtG* double mutant yielded strong signals with MenB-4C sera, again illustrating the inhibition of post-MenB-4C Ab binding to LOS by 3-lactose-HepII (**Figure 4A**), a finding also demonstrated with *N. gonorrhoeae* FA1090 and the FA1090*lgtG* mutant (**Figure 2C**).

#### 3.4.4 *N. gonorrhoeae* strain F62

*N. gonorrhoeae* F62 is reported to express a terminal GalNAc-modified LNT α-chain LOS (**Supplementary Table S2**) (48). Compatible with this structure, Western blots showed a higher MW LOS of WT F62 that did not react with mAbs 3F11, 2-1-L8, and 2C7 (**Figure 2B**). In addition to WT F62 LOS, we examined LOS of *lgtG*, *lgtG/lpt6*, *lpt3, lpt3/lpt6*, *lpt6*, *lgtA*, and *lgtA/lgtC* mutants (**Figure 4B**). The F62*lgtG* and the F62*lgtG/lpt6* mutants, but not the WT, initiated L1 LOS expression. In addition, the F62*lgtA* with a truncated α-chain was also positive for mAb L1. As expected, the L1 signal of the F62*lgtA* mutant disappeared when *lgtC* was also disrupted (**Figure 1A**). The F62*lgtA/lgtC* double mutant was reactive for both 2-1-L8 and 2C7, indicating a mixture of 3-PEA and 3-Gal-Glc on HepII. The appearance of 2C7 signals in the F62 mutants with a *lpt6* mutation (F62*lpt6* and F62*lpt3/lpt6*) correlated with that observed for FA19*lpt6*.

WT F62 LOS was recognized by nine of 10 of the post-MenB-4C sera (**Figure 2B**). The F62*lgtG*, F62*lgtG/lpt6*, and F62*lgtA* mutants expressing predominantly L1 LOS were weakly reactive with 19v5, while the F62*lgtA/lgtC* mutant producing L8 LOS with a 3-PEA-HepII showed stronger signals (**Figure 4B**). Mutations in *lpt6* became 2C7 positive and eliminated 19v5 recognition. Thus, accumulated data indicate that serum 19v5 reacted with LOS epitope(s) with either a 3- or 6-PEA but not glycosylated HepII. As anticipated, sera 7v5 reacted strongly with three F62 mutants expressing L1 LOS and did not bind mutants that were 2C7 positive (*lpt3/lpt6* and *lpt6*). Similarly, sera 17v5 displayed strong reactivity for three F62 mutants expressing L1 LOS and less reactivity with those expressing extended LNT α-chain LOS, regardless of HepII modifications (WT, *lpt3*, *lpt6*, and *lpt3/lpt6*).

### 3.5 Genomic content for inner core HepII modification genes

**Figure 5:**
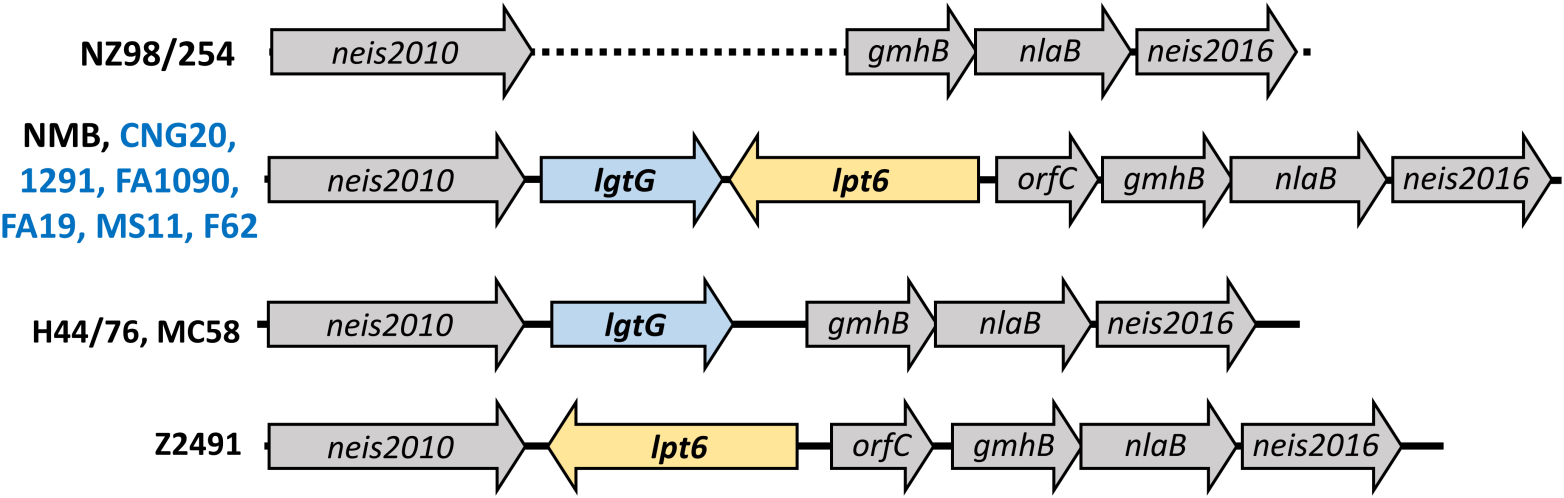
Schematic representation of the *lgtG-lpt6* genetic island. Four different genomic organizations are found in representative strains of *N. meningitidis* (black) or *N. gonorrhoeae* (blue). The dotted line indicated the absence of the *lgtG-lpt6* genetic island in strain NZ98/254.

As noted above, modifications on HepII by LgtG, Lpt3, and Lpt6 influenced the recognition by LOS-specific antibodies of post-MenB-4C sera. Of the HepII modification genes, *lpt3* is encoded separately from the *lgtG-lpt6* genetic island. Four configurations of the *lgtG-lpt6* locus are reported in *N. meningitidis* (1): *lgtG* alone (H44/76 and MC58) (2), *lpt6* alone (Z2491) (3), both *lgtG* and *lpt6* (NMB and all six *N. gonorrhoeae* strains in **Figure 2B**), and (4) missing the *lgtG-lpt6* genetic island (NZ98/254) (**Figure 5**). To more broadly evaluate the content of HepII modification genes in *N. gonorrhoeae* genomes, whole genome sequences (WGS) of *N. gonorrhoeae* in the PubMLST database were interrogated using the ‘Gene Presence analysis tool. Of 19,555 gonococcal genome records, 19,475 were marked as *lgtG*-positive (99.59%), and 80 were categorized as *lgtG*-negative. Blast analysis showed the majority of these 80 genomes (73/80), while having <50% sequence alignment, do contain highly homologous partial *lgtG* coding sequences (< 9 mismatches, and 0 - 1 gap) of variable lengths. The limited alignments were due to contig breaks within or adjacent to the *lgtG* coding sequence. One of the seven genomes without *lgtG* has poor WGS quality (> 1,000 contigs), and two genomes have contig breaks within the upstream neis2010 as well as between *lgtG* and *lpt6* or within *lpt6*. Thus, whether *lgtG* is present in these three genomes is unclear. Two genomes, while having large continuous contigs, lack the *lgtG-lpt6* island and the surrounding genes. Finally, two *lgtG*-negative genomes have the upstream neis2010 and the downstream *lpt6* genes, analogs to those of *N. meningitidis* strain Z2491 (**Figure 5**). Taken together, four of 19,552 genomes are most likely *lgtG* null (i.e., 99.98% *lgtG* positive). However, *lgtG* has a poly C track-causing phase onoff switch (the 11C track is in-frame). Overall, *lpt3* and *lpt6* were also encoded by most *N. gonorrhoeae*, with 98.95% and 99.82% prevalence, respectively, based on the Gene Presence analyses.

### 3.6 MenB-4C immunization produces LOS-specific IgG antibodies to gonococcal LOS that are bactericidal

**Figure 6:**
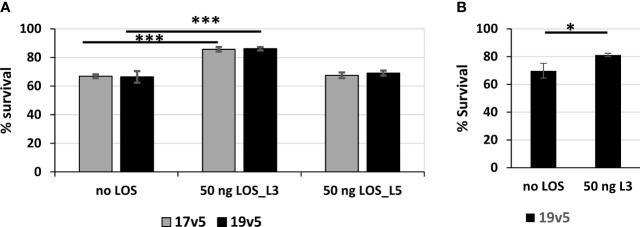
Serum bactericidal activities of post-MenB-4C sera against *N. gonorrhoeae*. **(A)** Serum bactericidal activities of two post-MenB-4C sera, 17v5 and 19v5, against *N. gonorrhoeae* strain 1291. When indicated, the reactions contain 50 ng of purified L3 LOS or L5 LOS. The CFU counts from reactions with 10% complement sera only are set as 100% for normalization. Data are represented as mean ± standard deviation. Statistical significance is analyzed by one-way ANOVA with an uncorrected Fishers least significant difference test, ^***^p < 0.001. **(B)** Serum bactericidal assays of sera 19v5 against *N. gonorrhoeae* strain FA1090, as described above. Statistical significance is analyzed by unpaired two-tailed Students *t*-tests, ^*^p < 0.05.

LOS antibodies have been reported to be bactericidal against *N. meningitidis* and *N. gonorrhoeae* (59–61). We estimated antibodies against LOS in post-MenB-4C accounted for ~10% of the total IgG response to gonococcal OMV preparations. The serum bactericidal activity (SBA) of post-MenB-4C sera against gonococcal strain 1291, which expresses L7 LOS (L3 LOS without terminal sialylation, **Table 1**) (46, 47) was determined. Two post-MenB-4C sera, 17v5 and 19v5, at 10% (v/v), yielded 33.2% ± 1.3% and 33.6% ± 4.0% killing of *N. gonorrhoeae* strain 1291, respectively (**Figure 6A**, no LOS). To remove antibodies specific to LOS, 50 ng of purified L3 LOS was added to the reactions. Similarly, equal amounts of L5 LOS, which does not react with the post-MenB-4C sera (**Figure 1C**), were examined in parallel as a control. The presence of excess L3 LOS, but not L5 LOS, significantly enhanced survival (*p* < 0.001) for both the 17v5 and 19v5 sera (**Figure 6A**), supporting that antibodies in these post-MenB-4C sera targeting LOS were bactericidal. The nonreactive L5 LOS at an equivalent amount and the presence of either purified LOS alone or the preimmune sera from the same individuals had no effect on survival. Sera 7v5 with immunodominant L1 LOS antibodies (**Figure 1C**) showed weaker bactericidal activity (13% ± 9.7%) against *N. gonorrhoeae* 1291, likely due to the absence of L1 LOS targets, and this serum was not examined with SBA assays. Excess L3 LOS also specifically titrated away the killing of the 19v5 serum against the 2C7-expressing *N. gonorrhoeae* strain FA1090 (**Figure 6B**), further confirming the contribution of LOS-specific bactericidal antibodies.

## 4 Discussion

The outer membrane of *N. meningitidis* and *N. gonorrhoeae*, containing proteins and lipooligosaccharides (LOS), is key to the immunological recognition of these pathogens. Much work has defined the differences and similarities between the *N. meningitidis* and *N. gonorrhoeae* outer membranes and has led to successful OMV vaccines for *N. meningitidis* (MenB-directed vaccines). A recent study has shown that OpcA and PorB are two important OMV protein antigens of MenB-4C, providing broad protection against different group B *N. meningitidis* (62). However, the identification of immunologically critical and distinctive gonococcal outer membrane antigens as vaccine candidates has continued to be elusive. Mounting evidence from multiple retrospective and ecological studies in several countries suggests that meningococcal OMV vaccines, including MenB-4C (Bexsero^R^), have effectiveness against gonococcal infections (4, 5, 7–10). The OMV proteins in MenB-4C share a high degree of amino acid sequence identity/similarity with corresponding homologs in *N. gonorrhoeae* (63, 64) and likely contribute to this effect, yet the precise mechanism(s) of cross-protection conferred by these meningococcal vaccines against *N. gonorrhoeae* remains to be determined.

LOS is the major surface component of *N. meningitidis* and *N. gonorrhoeae* outer membrane. LOS expression is heterogeneous, with multiple LOS species produced by a single strain. For example, studies using monoclonal antibodies indicate that a particular gonococcal strain can change LOS structure at high frequencies of 10^−2^10^−3^ due to slipped strand mispairing of LOS biosynthesis genes (65). Thus, considerable LOS antigenic variability exists in *N. meningitidis* and *N. gonorrhoeae*. Furthermore, several LOS glycans are not recognized as foreign and contribute to immune evasion by molecular mimicry with host antigen structures. For example, the α-chain lacto-*N*-neotetraose (Gal_β1→4_GlcNAc_β1→3_Gal_β1→4_Glc_β1→4_) is identical to human erythrocyte glycosphingolipids (66); and the alternative α-chain structure (Gal_α1→4_Gal_β1→4_Glc_β1→4_) of the L1 immunotype is structurally similar to human paraglobosides (67). Despite these challenges, LOS, as the vaccine antigen, has been evaluated for the prevention of both *N. meningitidis* (68) and *N. gonorrhoeae* (67), and unique conformational LOS-derived oligosaccharides are being examined as an *N. gonorrhoeae* vaccine candidates (69). In particular, the mAb 2C7-specific LOS epitope composed of a β-linked lactose (Gal-Glc) to HepI and an α-linked lactose to HepII (shaded in **Figure 1A**) (45, 61) is under active study. The 2C7 epitope is a conserved *N. gonorrhoeae* LOS inner core structure and is widely expressed by gonococci but not meningococci (70); in one study, mAb 2C7 recognized 95% of 101 second-passaged *N. gonorrhoeae* clinical isolates (45), and in a second, was present in 100% of the 75 isolates collected from Nanjing, China (71). The 3-Glc addition to HepII, a prerequisite for the 2C7 epitope, is added by LgtG, which is phase variable (49). As noted (and supported by this study, **Figure 2**), *N. gonorrhoeae* is the only member of the genus *Neisseria* that expresses lactose, extending from HepII (71). *N. meningitidis* strains (e.g., L2 strains such as NMB, **Figure 3A**) can place Glc at the 3-position of HepII, but further extension (i.e., 2C7 reactive) is not seen in *N. meningitidis* (**Figure 2A**).

**Figure 7:**
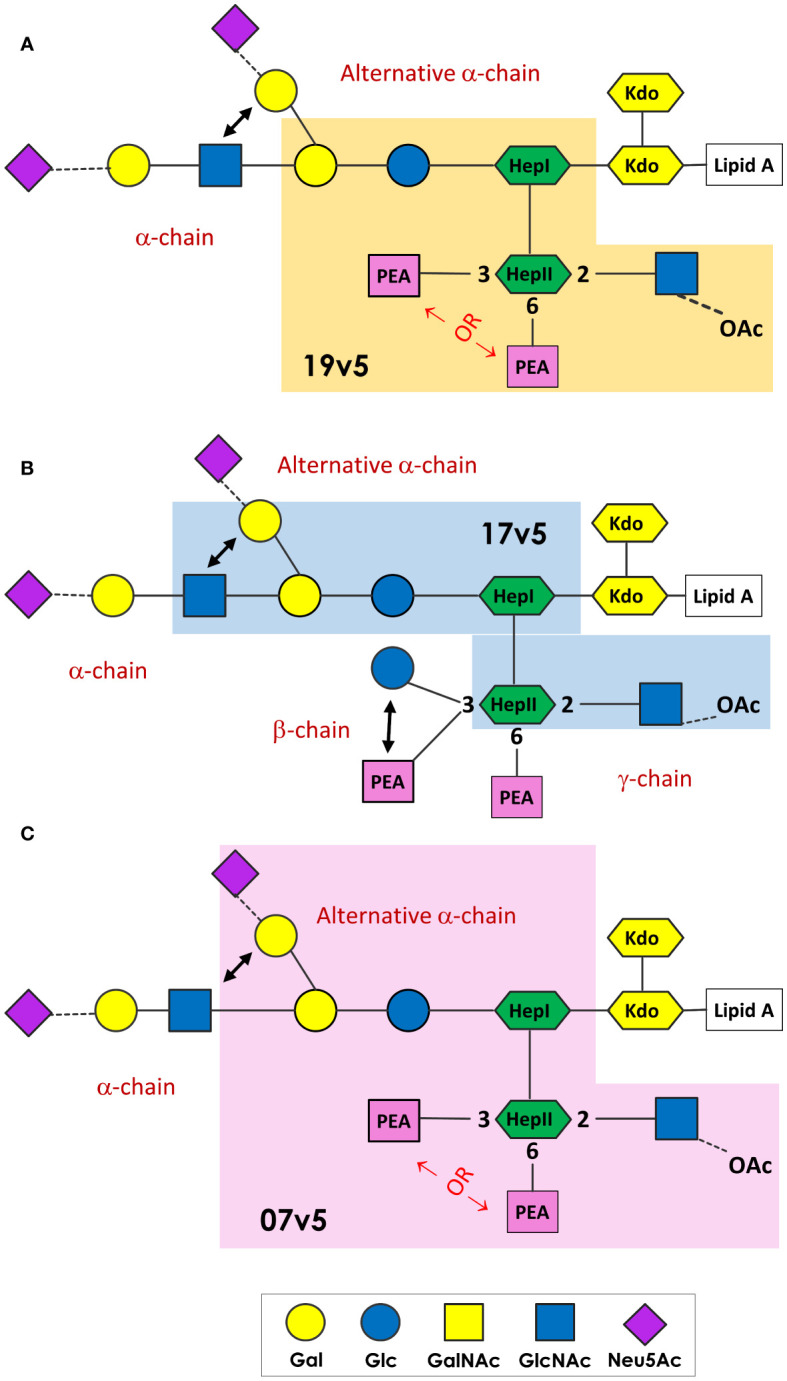
*N. meningitidis* and *N. gonorrhoeae* LOS epitopes recognized by post-MenB-4C sera. The minimal LOS structural epitope required for the dominant antibody population in 19v5 **(A)**, 7v5 **(B)**, and 17v5 **(C)** is shaded, and this description does not exclude some antibodies in the polyclonal population in these sera recognizing longer glycan structures. The key for the saccharide units is shown at the bottom.

MenB-4C immunization was found to elicit a spectrum of *N. meningitidis* and *N. gonorrhoeae* LOS-specific IgG antibodies. These LOS antibodies were in response to the L1 and L3 LOS antigens in MenB-4C, despite detergent extraction that reduces LOS content of 25 - 50% by weight relative to protein in native OMV to 5 - 8% residual LOS in MenB-4C (72). The immunodominant LOS Ab responses differed in vaccinated individuals. A schematic presentation summarizing the minimal LOS epitopes specific to predominant antibodies in three post-MenB-4C sera is shown in **Figure 7**. In sera 19v5, a conformational epitope that includes a truncated LNT or L1 α-chain (Gal-Glc-HepI) and a 2-GlcNAc-HepII with one PEA phosphorylation, but not 3-Glc, was recognized (**Figure 7A**). Other sera (e.g., 17v5) recognized a minimal tri-saccharide α-chain structure, including LOS immunotypes (L2, L3, L4, L7, L9) with complete LNT α-chain or alternate L1 α-chain, but not requiring specific HepII modifications, except the 2-GlcNAc-HepII (**Figure 7B**). The L6 LOS (GlcNAc-Gal-Glc-HepI) missing the terminal galactose of LNT remained quite reactive, but further truncation to an L8 structure (Gal-Glc-HepI) significantly diminished 17v5 recognition. Sialylation of the terminal Gal of the LNT or L1 was not required or blocked 17v5 Ab binding. In the 7v5 serum, L1-specific antibodies (Gal-Gal-Glc-HepI) were immunodominant. LOSs with a complete or truncated LNT α-chain were also weakly reactive with 7v5 if either a 3- or a 6-PEA-HepII was present and 3-Glc was absent on HepII (**Figure 7C**). An alternative α-chain linkage, maltose instead of lactose (e.g., L5, L10, and L11, **Table 1**) attached to HepI diminished or eliminated MenB-4C Ab reactivity. MenB-4C reactivity was also eliminated or diminished in the presence of the Gal-Glc α-linked to HepII, needed for 2C7 reactivity. The *lgtG* mutation in multiple *N. meningitidis* and *N. gonorrhoeae* strains enhanced recognition by post-MenB-4C sera (**Figures 2**, **3**, **7**). Thus, the 3-Glc or 3-Gal-Glc addition on HepII was an unfavorable feature for LOS antibodies elicited by MenB-4C. The LOS in MenB-4C OMVs are prepared from strain NZ98/254, which does not encode *lgtG* (**Figure 5**) and thus lacks a 3-Glc-HepII.

LOS-specific antibodies of post-MenB-4C sera were bactericidal against gonococci (**Figure 6**). Killing of gonococcal strain 1291 expressing the L7 LOS (**Table 1**) can be titrated away by excess purified L3 LOS, but not by a nonbinding L5 LOS (**Figure 1C**) that has a Gal_β1→4_GlcNAc_β1→3_Gal_β1→4_Glc_β1→4_Glc_β1→4_-α-chain with a 3-Glc addition at HepII. Post-MenB-4C sera 17v5 and 19v5 at 10% (v/v) with a 10% complement source showed ~30% killing against the *N. gonorrhoeae* strain 1291 and, as anticipated, near 100% killing of the vaccine strain NZ98/254 (unpublished data). Gonococci lack the MenB-4C-targeted protein antigens present in meningococci (e.g., NadA, FHbp, and PorA), and LOS-specific antibodies constitute a small fraction of total antibodies (~10% estimated in **Figure 1B**). However, the reduction in killing by post-MenB-4C sera 17v5 and 19v5 in the presence of excess L3 LOS suggests that the LOS-specific antibodies represent a significant proportion of bactericidal antibodies against *N. gonorrhoeae* in these sera. In separate preliminary studies, a significant amount of the remaining *N. gonorrhoeae* bactericidal activity in serum 17v5 is directed at the cross-reactive *N. gonorrhoeae* OMV proteins, especially NHBA (unpublished data).

Inner core structural variations of *N. meningitidis* and *N. gonorrhoeae*, LOS affect susceptibility to Ab-mediated immunity. In previous work on *N. meningitidis* and *N. gonorrhoeae*, human sera, and functional mAbs were shown to recognize LOS inner core heptose structures (34, 73, 74). For example, mAb B5, which specifically targets the 3-PEA-HepII of *N. meningitidis* LOS exhibits opsonophagocytic and bactericidal killing against the MC58*::galE* mutant, and a mutation in *lpt3* resulted in a significant decrease in the functional activity of mAb B5 (34). Thus, the 3-PEA-HepII has been proposed to be an *N. meningitidis* immunodominant epitope (34) and is found on ~70% of hypervirulent *N. meningitidis* strains (73). Furthermore, Ram et al. showed that the PEA groups on *N. meningitidis* and *N. gonorrhoeae* HepII form amide linkages with C4b (75). Strains with 6-PEA are more efficient than those with 3-PEA in binding C4b and are more susceptible to complement-mediated killing in serum bactericidal assays (75); this correlates with the dominance of 3-PEA-bearing meningococci among clinical *N. meningitidis* isolates. In addition, mAb 2C7, which requires 3-lactose-HepII for binding, showed functional activity against all gonococcal HepI LOS structures defined by various *lgtA/C/D* on/off combinations (76, 77). The data presented in this paper show that HepII inner core modifications by PEA or glucose/lactose greatly influence recognition by antibodies elicited by MenB-4C immunization. The preference for 3-PEA-HepII over a glycosylated HepII was the likely result of the 3-PEA-HepII LOS antigen present in the OMV component of MenB-4C.

Convalescent infections and multicomponent vaccines such as MenB-4C induce antibodies (78), including those to LOS that act cooperatively and synergistically, as simultaneous binding of antibodies to various surface-exposed antigens can overcome the threshold density of antigen - antibody complexes needed for complement activation (79). Previous studies of OMV-based meningococcal vaccines have demonstrated that LOS antibodies significantly contribute to the bactericidal activity against *N. meningitidis* in vaccine responders (80, 81). Furthermore, antibodies to LOS are found to mediate significant bactericidal activity in normal human sera against group B meningococci using bactericidal depletion assays, with the implication that anti-LOS antibodies can persist and play an important role in natural immunity to *N. meningitidis* (82). In support of the importance of *N. gonorrhoeae* LOS antibodies, an early study by Rice et al. (83) noted that specific inhibition of serum bactericidal activity against the infecting disseminated gonococcal strain was detected when the patients convalescent serum was pre-incubated with the endotoxin fraction and the outer membrane, while the protein fraction exhibited no inhibition. Antibodies to *N. gonorrhoeae* LOS have been found in both normal human sera and in sera from *N. gonorrhoeae-*infected individuals (78). Furthermore, experimental gonococcal urethritis and reinfection with homologous gonococci in male volunteers show that men with IgG antibodies against LOS were significantly less likely to become infected when later challenged than were men who did not have an IgG response to LOS (84).

Thus, MenB-4C immunization elicits serum bactericidal IgG antibodies to shared LOS conformational epitopes on *N. gonorrhoeae* and *N. meningitidis* that may contribute to the effectiveness of meningococcal OMV vaccines against gonococcal infections. The LOS immunogenicity of MenB-4C was anchored in the prokaryotic heptoses of LOS, a sugar unique to gram-negative bacteria and not found in human antigens (85). When a microarray of the α-chain glycans (L1, L6, L8, and LNT) not linked to heptose was examined, no differential binding by pre- or post-MenB-4C sera was observed (unpublished data). LOS-specific recognition was influenced by heterogeneous LOS expression in *N. meningitidis* and *N. gonorrhoeae*, and MenB-4C-induced LOS Ab populations were strikingly different among vaccinated individuals. The individual variability in response to LOS may suggest vaccine boosting of preexisting populations of glycan-specific memory B cells (86) previously programmed following exposure to *Neisseria* spp. LOS antigens. LOS antibodies in many post-MenB-4C sera recognized gonococci without the 2C7 epitope. The study suggests that modification of the LOS structures in licensed meningococcal OMV vaccines may enhance protection against *N. gonorrhoeae*. Because the 2C7 LOS epitope is unique to gonococci, the inclusion of the 2C7-reactive oligosaccharides (or 2C7-positive *N. gonorrhoeae* OMV) as an example in such tailored next-generation vaccine formulations may enhance the contribution of LOS-specific antibodies and increase the effectiveness of OMV vaccines against *N. gonorrhoeae*.

## Data availability statement

The raw data supporting the conclusions of this article will be made available by the authors without undue reservation.

## Ethics statement

The studies involving humans were approved by National Research Ethics Service, Wandsworth Research Ethics Committee. The EudraCT number was 2008-007182-23. The studies were conducted in accordance with the local legislation and institutional requirements. The participants provided their written informed consent to participate in this study.

## Author contributions

Y-LT: Conceptualization, Formal analysis, Funding acquisition, Investigation, Methodology, Project administration, Supervision, Visualization, Writing – original draft, Writing – review & editing. SS: Data curation, Investigation, Writing – review & editing. RB: Formal analysis, Resources, Writing – review & editing. DS: Conceptualization, Funding acquisition, Supervision, Writing – original draft, Writing – review & editing.

## Supplementary material

Supplementary Figure 1 Western blots probed with pre-absorbed 19v5 sera.
