# Supplementary material for: Neisseria gonorrhoeae lipooligosaccharide glycan epitopes recognized by bactericidal IgG antibodies elicited by the meningococcal group B-directed vaccine, MenB-4C
Source: Front Immunol. 2024 Feb 19;15:1350344. doi: 10.3389/fimmu.2024.1350344 (PMC10909805; doi:10.3389/fimmu.2024.1350344)
Supplement: Supplementary file 1 [file Table_1.docx]

**Table S1. Strains used in this study**

| **Strains** | **Genotype** | **Reference** |
| --- | --- | --- |
| 126E | *N. meningitidis* L1 LOS immunotyping strain | Laboratory collection |
| 35E | *N. meningitidis* L2 LOS immunotyping strain | Laboratory collection |
| 6275 | *N. meningitidis* L3 LOS immunotyping strain | Laboratory collection |
| 89I | *N. meningitidis* L4 LOS immunotyping strain | Laboratory collection |
| M981 | *N. meningitidis* L5 LOS immunotyping strain | Laboratory collection |
| M992 | *N. meningitidis* L6 LOS immunotyping strain | Laboratory collection |
| 6155 | *N. meningitidis* L7 LOS immunotyping strain | Laboratory collection |
| M978 | *N. meningitidis* L8 LOS immunotyping strain | Laboratory collection |
| 120M | *N. meningitidis* L9 LOS immunotyping strain | Laboratory collection |
| 7880 | *N. meningitidis* L10 LOS immunotyping strain | Laboratory collection |
| 7889 | *N. meningitidis* L11 LOS immunotyping strain | Laboratory collection |
| 7897 | *N. meningitidis* L12 LOS immunotyping strain | Laboratory collection |
| NMB | Wild type group B *N. meningitidis* | (1) |
| NMB*cssA* | NMB with *cssA::tetM* | (1) |
| NMB*lgtG* | NMB with *lgtG::aphA3* | (2) |
| NMB*lpt3* | NMB with *lpt3::aphA3* | (3) |
| NMB*lpt6* | NMB with *lpt6::erm* | (3) |
| NMB*lgtG/lpt6* | NMB*lpt6* with *lgtG::aphA3* | This study |
| NMB*lgtA* | NMB with *lgtA::aphA3* | This study |
| NMB*lgtF* | NMB with *lgtF::Ω(Spec)* | This study |
| NMB*galE* | NMB with *galE::tetM* | (4) |
| NMB*galE*/*lgtG* | NMB*galE* with *lgtG::aphA3* | This study |
| NMB*rfaK* | NMB with *rfaK::Ω(Spec)* | (5) |
| NZ98/254 | Wild type group B *N. meningitidis* | Laboratory collection |
| NZ98*cssA* | NZ98/254 with *cssA::tetM* | This study |
| NZ98*lpt3* | NZ98/254 with *lpt3::aphA3* | This study |
| NZ98*lgtA/lgtC* | NZ98/254 with *lgtA/C::aphA3* | This study |
| NZ98*rfaK* | NZ98/254 with *rfaK::Ω(Spec)* | This study |
| H44/76 | Wild type group B *N. meningitidis* | Laboratory collection |
| CNG20 | Wild type *N. gonorrhoeae* | (6) |
| 1291 | Wild type *N. gonorrhoeae* | Laboratory collection |
| MS11 | Wild type *N. gonorrhoeae* | Laboratory collection |
| FA1090 | Wild type *N. gonorrhoeae* | (7) |
| FA1090lgtG | FA19 with *lgtG::aphA3* | (7) |
| FA19 | Wild type *N. gonorrhoeae* | Laboratory collection |
| FA19*lgtG* | FA19 with *lgtG::aphA3* | Laboratory collection |
| FA19*lpt3* | FA19 with *lpt3::aphA3* | Laboratory collection |
| FA19*lpt6* | FA19 with *lpt6::erm* | Laboratory collection |
| FA19*lgtG/lpt6* | FA19 with *lgtG::aphA3* & *lpt6::erm* | Laboratory collection |
| F62 | Wild type *N. gonorrhoeae* | Laboratory collection |
| F62*lgtA* | F62 with *lgtA::aphA3* | (8) |
| F62*lgtA/lgtC* | F62 with *lgtA::aphA3* & *lgtC::erm* | Laboratory collection |
| F62*lgtG* | F62 with *lgtG::aphA3* | Laboratory collection |
| F62*lpt3* | F62 with *lpt3::aphA3* | Laboratory collection |

**Table S2. LOS structures of *Ng* reference strains**

| **Strain** | **α-chain (HepI)** | **β-chain (HepII)** | | **References** |
| --- | --- | --- | --- | --- |
|  |  | **3-** | **6-** |  |
| 1291 | Gal-GlcNAc-Gal-Glc- | PEA | nd | (9, 10) |
| FA1090^a^ | Gal-GlcNAc-Gal-Glc- | Gal-Glc | PEA | NA |
| MS11 | Gal-GlcNAc- Gal-GlcNAc-Gal-Glc-  GalNAc- Gal-GlcNAc-Gal-Glc-  Gal-GlcNAc-Gal-Glc-  GlcNAc-Gal-Glc-  (L1) Gal-Gal-Glc- | Gal-Glc; PEA^b^ | PEA_(0-1)_ | (11, 12) |
| FA19^c^ | Gal-GlcNAc-Gal-Glc-  Gal-Glc-  (L1) Gal-Gal-Glc- | PEA^b^ | PEA | (9, 13) |
| F62 | GalNAc- Gal-GlcNAc-Gal-Glc-  Gal-GlcNAc-Gal-Glc- | PEA^b^ | nd | (14) |

a, FA1090 structure predicted by mAb reactivities.

b, F62, MS11 and FA19 were reported to be positive for mAb B5, which recognizes 3-PEA-HepII (15).

c, FA19 is reported to express both L8-like and L3-like LOS structures by Burch et al (9); however, the FA19 strain used in this study produces predominantly L8-like LOS structure as indicated by Shafer et al (13). In addition, Western blot data (Fig. 8) with mAb L1 indicated the presence of L1 LOS in WT FA19.

nd, not determined.

**Table S3. LOS structures of meningococcal group B strain NMB and mutants.**

| **Strains** | **α-chain (Hep I)** | **β-chain (HepII)** | | **γ-chain (HepII)** | **Reference** |
| --- | --- | --- | --- | --- | --- |
|  |  | **3-** | **6-** | **2-** |  |
| **NMB** | Neu5Ac-Gal-GlcNAc-Gal-Glc- | Glc | PEA | GlcNAc(OAc) | (3, 16) |
| ***lgtG*** | Neu5Ac-Gal-GlcNAc-Gal-Glc- | H | PEA | GlcNAc(OAc) | (2) |
| ***lpt3*** | Neu5Ac-Gal-GlcNAc-Gal-Glc- | Glc | PEA | GlcNAc(OAc) | (3) |
| ***lpt6*** | Neu5Ac-Gal-GlcNAc-Gal-Glc- | Glc | H | GlcNAc(OAc) | (3) |
| ***lpt6/lgtG*** | Neu5Ac-Gal-GlcNAc-Gal-Glc- | PEA | H | GlcNAc(OAc) | na |
| ***lgtA*** | Gal-Glc- | Glc^a^ | PEA | GlcNAc(OAc) | na |
| ***lgtF*** | H | H | PEA | GlcNAc(OAc)^c^ | (4) |
| ***rfaK*** | H | PEA | PEA | H | (17) |
| ***galE*** | Glc_(1-3)_-Glc | Glc | PEA | GlcNAc(OAc)^c^ | (3, 4) |
| ***galE/lgtG*** | Glc_(1-3)_-Glc | PEA^b^ | PEA | GlcNAc(OAc)^c^ | na |
| ***cssA*** | Gal-GlcNAc-Gal-Glc- | Glc | PEA | GlcNAc(OAc) | (18) |

White cells: structures previously confirmed biochemically. Shaded cells: predicted structures based on the profiles of mAb reactivities.

a. The *lgtA* mutant has a 3-Glc-HepII because there is no signal for 2-1-L8 that recognizes Gal-Glc-HepI with 3-PEA.

b. 3-PEA was predicted for the *galE/lgtG* mutant based on the lack of LgtG function in the mutant expressing a truncated α-chain structure (3).

c. The gene, *lot3*, encoding the O-acetyltransferase of 2-GlcNAc on HepII is functional in strain NMB. Thus, the GlcNAc of these mutants are predicted to be O-acetylated (19); however, there is no biochemical structure determination.

1. Swartley JS, Stephens DS. Identification of a genetic locus involved in the biosynthesis of N-acetyl-D-mannosamine, a precursor of the (α 2-->8)-linked polysialic acid capsule of serogroup B *Neisseria meningitidis*. *J Bacteriol* (1994) 176:1530-4. doi:

2. Tzeng YL, Datta A, Ambrose KD, Davies JK, Carlson RW, Stephens DS, et al. The MisR/MisS two-component regulatory system influences inner core structure and immunotype of lipooligosaccharide in *Neisseria meningitidis*. *J Biol Chem* (2004) 279:35053-62. doi: 10.1074/jbc.M401433200

3. Kahler CM, Datta A, Tzeng YL, Carlson RW, Stephens DS. Inner core assembly and structure of the lipooligosaccharide of *Neisseria meningitidis*: capacity of strain NMB to express all known immunotype epitopes. *Glycobiology* (2005) 15:409-19. doi: 10.1093/glycob/cwi018

4. Lee FK, Stephens DS, Gibson BW, Engstrom JJ, Zhou D, Apicella MA. Microheterogeneity of *Neisseria* lipooligosaccharide: analysis of a UDP-glucose 4-epimerase mutant of *Neisseria meningitidis NMB*. *Infect Immun* (1995) 63:2508-15. doi: 10.1128/iai.63.7.2508-2515.1995

5. Kahler CM, Carlson RW, Rahman MM, Martin LE, Stephens DS. Inner core biosynthesis of lipooligosaccharide (LOS) in *Neisseria meningitidis* serogroup B: identification and role in LOS assembly of the alpha1,2 N-acetylglucosamine transferase (RfaK). *J Bacteriol* (1996) 178:1265-73. doi: 10.1128/jb.178.5.1265-1273.1996

6. Tzeng YL, Bazan JA, Turner AN, Wang X, Retchless AC, Read TD, et al. Emergence of a new *Neisseria meningitidis* clonal complex 11 lineage 11.2 clade as an effective urogenital pathogen. *Proc Natl Acad Sci U S A* (2017) 114:4237-42. doi: 10.1073/pnas.1620971114

7. Gulati S, Zheng B, Reed GW, Su X, Cox AD, St Michael F, et al. Immunization against a saccharide epitope accelerates clearance of experimental gonococcal infection. *PLoS Pathog* (2013) 9:e1003559. doi: 10.1371/journal.ppat.1003559

8. Balthazar JT, Gusa A, Martin LE, Choudhury B, Carlson R, Shafer WM. Lipooligosaccharide Structure is an Important Determinant in the Resistance of *Neisseria Gonorrhoeae* to Antimicrobial Agents of Innate Host Defense. *Front Microbiol* (2011) 2:30. doi: 10.3389/fmicb.2011.00030

9. Burch CL, Danaher RJ, Stein DC. Antigenic variation in *Neisseria gonorrhoeae*: production of multiple lipooligosaccharides. *J Bacteriol* (1997) 179:982-6. doi: 10.1128/jb.179.3.982-986.1997

10. John CM, Griffiss JM, Apicella MA, Mandrell RE, Gibson BW. The structural basis for pyocin resistance in *Neisseria gonorrhoeae* lipooligosaccharides. *J Biol Chem* (1991) 266:19303-11. doi:

11. Schneider H, Griffiss JM, Boslego JW, Hitchcock PJ, Zahos KM, Apicella MA. Expression of paragloboside-like lipooligosaccharides may be a necessary component of gonococcal pathogenesis in men. *J Exp Med* (1991) 174:1601-5. doi: 10.1084/jem.174.6.1601

12. John CM, Schneider H, Griffiss JM. *Neisseria gonorrhoeae* that infect men have lipooligosaccharides with terminal N-acetyllactosamine repeats. *J Biol Chem* (1999) 274:1017-25. doi: 10.1074/jbc.274.2.1017

13. Shafer WM, Datta A, Kolli VS, Rahman MM, Balthazar JT, Martin LE, et al. Phase variable changes in genes *lgtA* and *lgtC* within the *lgtABCDE* operon of *Neisseria gonorrhoeae* can modulate gonococcal susceptibility to normal human serum. *J Endotoxin Res* (2002) 8:47-58. doi:

14. Yamasaki R, Bacon BE, Nasholds W, Schneider H, Griffiss JM. Structural determination of oligosaccharides derived from lipooligosaccharide of *Neisseria gonorrhoeae* F62 by chemical, enzymatic, and two-dimensional NMR methods. *Biochemistry* (1991) 30:10566-75. doi: 10.1021/bi00107a028

15. Mackinnon FG, Cox AD, Plested JS, Tang CM, Makepeace K, Coull PA, et al. Identification of a gene (*lpt-3*) required for the addition of phosphoethanolamine to the lipopolysaccharide inner core of *Neisseria meningitidis* and its role in mediating susceptibility to bactericidal killing and opsonophagocytosis. *Mol Microbiol* (2002) 43:931-43. doi: 10.1046/j.1365-2958.2002.02754.x

16. Rahman MM, Stephens DS, Kahler CM, Glushka J, Carlson RW. The lipooligosaccharide (LOS) of *Neisseria meningitidis* serogroup B strain NMB contains L2, L3, and novel oligosaccharides, and lacks the lipid-A 4'-phosphate substituent. *Carbohydr Res* (1998) 307:311-24. doi: 10.1016/s0008-6215(98)00012-3

17. Rahman MM, Kahler CM, Stephens DS, Carlson RW. The structure of the lipooligosaccharide (LOS) from the alpha-1,2-N-acetyl glucosamine transferase (rfaK(NMB)) mutant strain CMK1 of *Neisseria meningitidis*: implications for LOS inner core assembly and LOS-based vaccines. *Glycobiology* (2001) 11:703-9. doi: 10.1093/glycob/11.8.703

18. Kahler CM, Martin LE, Shih GC, Rahman MM, Carlson RW, Stephens DS. The (α2-->8)-linked polysialic acid capsule and lipooligosaccharide structure both contribute to the ability of serogroup B *Neisseria meningitidis* to resist the bactericidal activity of normal human serum. *Infect Immun* (1998) 66:5939-47. doi: 10.1128/IAI.66.12.5939-5947.1998

19. Kahler CM, Lyons-Schindler S, Choudhury B, Glushka J, Carlson RW, Stephens DS. O-Acetylation of the terminal N-acetylglucosamine of the lipooligosaccharide inner core in *Neisseria meningitidis*. Influence on inner core structure and assembly. *J Biol Chem* (2006) 281:19939-48. doi: 10.1074/jbc.M601308200
