# Supplementary figures and images for: Neisseria gonorrhoeae lipooligosaccharide glycan epitopes recognized by bactericidal IgG antibodies elicited by the meningococcal group B-directed vaccine, MenB-4C
Source: Front Immunol. 2024 Feb 19;15:1350344. doi: 10.3389/fimmu.2024.1350344 (PMC10909805; doi:10.3389/fimmu.2024.1350344)

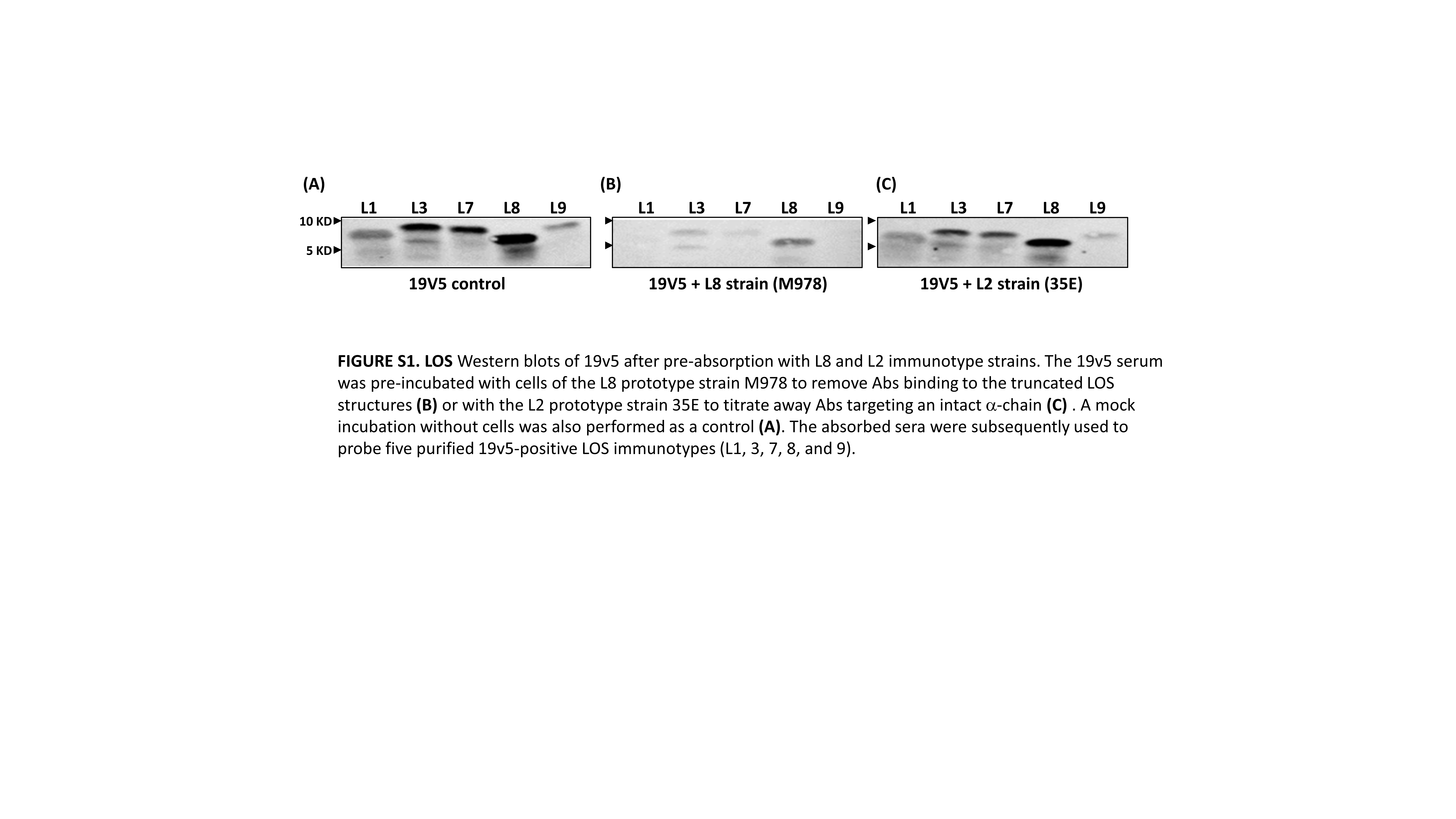

Supplement: Supplementary Figure 1 — Western blots probed with pre-absorbed 19v5 sera. [file Image_1.tif]
